# Analysis of Size-Dependent Linear Static Bending, Buckling, and Free Vibration Based on a Modified Couple Stress Theory

**DOI:** 10.3390/ma15217583

**Published:** 2022-10-28

**Authors:** Feixiang Tang, Siyu He, Shaonan Shi, Shun Xue, Fang Dong, Sheng Liu

**Affiliations:** 1 Key Laboratory of Transients in Hydraulic Machinery, Ministry of Education, Wuhan University, Wuhan 430072, China; 2 The Institute of Technological Sciences, Wuhan University, Wuhan 430072, China

**Keywords:** Kirchhoff plate theory, modified couple stress theory, scale effect, buckling, free vibration

## Abstract

The purposes of this paper are to study bending, buckling, and vibration by considering micro-scale effects using the Kirchhoff thin-plate theory and to consider small deflections, neglecting higher-order nonlinear terms. The governing equations for the bending, buckling, and vibration of the system are obtained using the equilibrium method coupled with the Kirchhoff thin-plate theory and a modified couple stress theory (MCST). The concept of the equivalent bending stiffness (EBS) of micro-thin plates is proposed to describe the scale effect. The Navier method is used to obtain analytical solutions for the bending, buckling, and free vibration of thin plates under simply supported boundary conditions with scale effects. The numerical results are presented to investigate the influence of scale effects on deflection, critical buckling load, buckling topography, and thin-plate natural frequency. The results show that the scale effect increases the equivalent stiffness of the thin plate, which leads to a decrease in deflection, a larger critical buckling load, and an increase in natural frequency, but does not affect the buckling topography. The MSCT is invalid when the thickness is greater than 10 times the scale effect parameter, thus defining the scope of application of the scale effect. This research study may contribute to the design of micro-scale devices such as MEMSs/NEMSs.

## 1. Introduction

In recent years, research on micro-electromechanical systems and nano-electromechanical systems has increased. Due to their small volume, light weight, low energy consumption, fast response, and excellent performance, MEMS/NEMS devices play an irreplaceable role in some high-precision fields, including electronics, machinery, materials, automatic control, physics, chemistry, biology, and other disciplines. This technology has shown a very wide range of applications in consumer electronics, industrial control, medical science and technology, communication, national defense, and other aspects [1,2,3]. The manufacturing sensitivity and reliability issues of multi-field and multi-scale coupling are challenges for these devices [4,5,6,7]. However, many experimental phenomena [8,9,10] indicate that when the size of the material structure is reduced to the micro–nano levels, its mechanic behavior is very different from that on the macro scale, usually with a large surface-area-to-volume ratio and obvious scale effects and surface effects. In this case, the traditional theory of the continuum model can no longer explain this phenomenon accurately on the micro scale. To meet the needs of theoretical research and scientific development, many theories, including characteristic scale parameters, have been proposed. Common micro-scale theories include the nonlocal theory, the couple stress theory, the strain gradient theory, the surface elasticity theory, etc. Among them, the nonlocal theory [11,12] considers the global effect caused by the long-range action of the micro structure. The strain gradient theory [13] discusses the influence of higher-order strain tensors on the structure. The couple stress theory [14] takes into account the rotational degrees of freedom of materials during deformation. In addition, the surface elasticity theory [15] can effectively characterize the influence of surface free energy on the mechanical behavior of materials. These theories make up for the limitation of the traditional continuum theory to a certain extent through different micro-mechanical mechanisms and have been successfully used by many scholars for various experimental analyses and exploration.

Recently, Ahmad Reza Khorshidvand et al. [16] investigated the vibration behavior of porous FG circular plates using the classical, and the first- and third-order shear deformation plate theories. The effect of porosity, pore distribution, and other factors on plate vibration were considered. Saeed Amir et al. [17] used the modified strain gradient theory and the first-order shear deformation theory to consider the bending, buckling, and free vibration behavior of porous nano-composite micro plates under wet thermal–mechanical loading. Based on the nonlocal strain gradient theory, Yan et al. [18] discussed the application and dynamic behavior of carbon nano tubes as fluid nano sensors and studied the effects on the frequency caused by the fluid, the moment of inertia, non-classical boundary conditions, vibration modes, nonlocal parameters, and strain gradient parameters. The results showed that with the increase in strain gradient and nonlocal parameters, the fluid nano sensors exhibited a stiffness-softening effect. Saeed Amir et al. [19] studied the vibration of porous rectangular plates based on the shear deformation theory and the nonlocal theory. The results of the study contributed to the design of more efficient actuators and sensors. Meanwhile, Phung-van P. et al. [20] studied the nonlinear bending behavior of nano-porous metal foam plates using the nonlocal strain gradient theory and found that the stiffness of the metal foam plates softened with the increase in the nonlocal parameters. These findings may play an important role in the design of metal foam structures. Eltaher et al. [21] studied the vibration and buckling behavior of FG nano beams using the nonlocal strain gradient theory. The established model can be used as a benchmark for analyzing the mechanical behavior of nano beams in a thermomagnetic field. The traditional theoretical model of couple stress contains many material-related scale parameters. Cao and Tucker [22] established the nonlinear partial differential equation for the movement of slender elastic rods based on the couple stress theory. The traditional couple stress theory is difficult to be applied in engineering because of many material size parameters, so a modified couple stress theory was developed. In 2002, Yang et al. [23] proposed a modified couple stress theory that requires only one scale parameter, which greatly reduces the difficulty in solving the problem. Ehsan Alheid et al. [24] studied the vibration behavior of porous bending micro beams using the higher-order shear deformation theory and the modified coupled stress theory. The results showed that the material properties of the porous core and nano-composite panels had a significant effect on the vibration response of the model. Mahdi Bodaghi et al. [25] studied the free vibration behavior of a functional gradient, porous cylindrical shell using the sinusoidal theory and the modified coupled stress theory. The results of the study can be used to design and fabricate more reliable micro-cylindrical structures in thermodynamic environments. Saeed Amir et al. [26] investigated the thermal buckling behavior of circular/annular functionally graded graphene nano platelet (GNP)-reinforced porous nano composites. The results of this study contributed to the design and creation of engineered structures with improved performance. Mousavi et al. [27] combined the triangular shear deformation theory and the MCST to study the free vibration behavior of porous micro (PM) beams. It was found that while increasing the porosity decreased the natural frequency, increasing the volume fraction of carbon nano tubes (CNTs) increased the natural frequency. Saeed Amir et al. [28] studied the mechanical behavior of porous sandwich structures under magnetic field forces based on the MSCT and the higher-order shear deformation theory. In 2011, Chen et al. [29] proposed a new modified couple stress theory applicable to anisotropic materials. Now, based on the modified couple stress theory, Li et al. [30] studied the free vibration of one-dimensional piezoelectric quasicrystal micro beams and developed various shear displacement models of piezoelectric quasicrystal micro beams. Based on the new modified couple stress theory, Zhang et al. [31] established the damp–heat stability model of Mindlin laminate under the combined action of mechanical and damp–heat loads. Therefore, it is of great practical value to study the effect of the scale effect on the mechanical behavior of micro–nano structures. From the above, it is clear that the MCST is more easily used in engineering practice than the modified strain gradient theory (MSGT) and the nonlocal strain gradient theory (NSGT) due to considering only one scale parameter and is useful for the design of micro-scale devices such as MEMSs/NEMSs.

MEMS/NEMS device structures are mainly micro beams, micro plates, and micro shells [32]. Machining and installation often cause geometric defects or residual stresses due to the micro size. They have different mechanical properties and scale effects, and large deformation compared with macro-scale structures [33]. Eltaher et al. [34] studied the mechanical behavior of carbon nano tubes under dynamic loading, and the established model can be used for the design of MEMSs/NEMSs. The scope of the application of scale effect parameters and theories often differs for different materials. The study of the integration of materials, structures, and micro-scale theories can guide the design, fabrication, and application of structural unit devices such as micro-cantilever beams and micro-thin plates. So, the work of size-dependent research is necessary and significant.

The literature review summarizes some important works on the scale effect theory, such as the nonlocal strain gradient theory, the couple stress theory, the modified couple stress theory, etc. The principle of minimum potential energy in the related literature is often used to solve buckling and bending problems. However, the equilibrium method is rarely used to solve the problem. This paper’s conjunction of the Kirchhoff thin-plate theory and the modified couple stress theory, compared with most authors who use the minimum potential energy principle, innovatively uses the equilibrium method to obtain the bending, buckling, and free vibration control equations of the system. Analytical solutions for the bending, buckling, and vibration of micro-scale thin plates are obtained. The proposed EBS model can easily convert the classical theory (CT) to the MCST. The influence of the scale effect on equivalent bending stiffness, deflection, critical buckling load, and buckling topography is investigated. The highlight of this article is to obtain the size range of the micro-scale theory, which is consistent with the experiment [35]. The experiment shows that the mechanical behavior of materials on the micron scale is size dependent. The classical theory is invalid when the size reaches the micro–nano scales, while the coupling theory of the Kirchhoff thin-plate theory and the scale effect theory considering small deflection and ignoring high-order nonlinear terms is of great significance. It is sometimes hard to obtain the results using nonlinear analyses, while with linear analyses, it is easy to obtain solutions that could be useful in some practical engineering problems. The Navier method is utilized to obtain bending, buckling, and free vibration analytical solutions. The proposed model can be used for the design and analysis of MEMSs/NEMSs.

## 2. Theories and Methods

**Figure 1:**
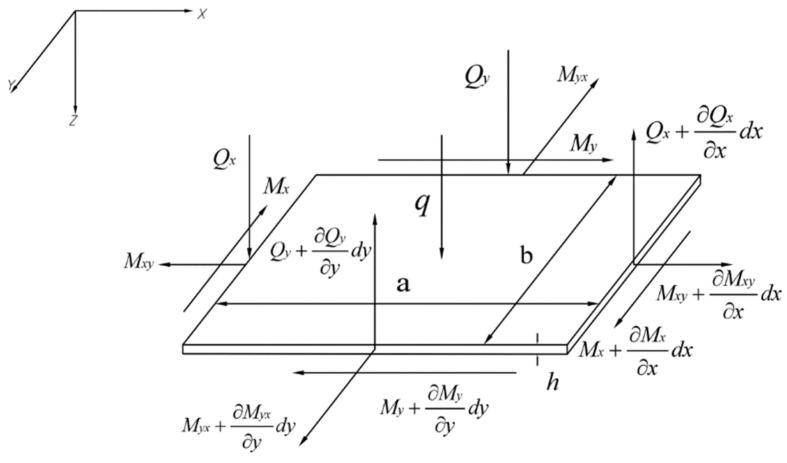
Loading and geometry of a thin, rectangular micro-plate model.

The Kirchhoff thin-plate theory is used to resolve the bending, buckling, and free vibration problem. Figure 1 shows an isotropic thin plate with length a, width b, and thickness h under external lateral load q.

The equation of motion of the isotropic thin plate based on the modified couple stress theory [23] and the Kirchhoff thin-plate theory is expressed as [36]:

(1) $$\sigma_{ij}=\lambda \varepsilon_{kk}\delta_{ij}+2\mu \varepsilon_{ij}$$

(2) $$\varepsilon_{ij}=\frac{1}{2}(u_{i,j}+u_{j,i})$$

(3) $$\chi_{ij}=\frac{1}{2}(\theta_{i,j}+\theta_{j,i})$$

(4) $$\theta_{i}=\frac{1}{2}e_{ijk}u_{k,j}$$

(5) $$m_{ij}=2l^{2}\mu \chi_{ij}$$

(6) $$\mu =G=\frac{E}{2\left(1+\upsilon \right)}$$

(7) $$\lambda =\frac{E\upsilon }{\left(1+\upsilon \right)\left(1-2\upsilon \right)}$$

where *σ_ij_*, *ε_ij_*, *χ_ij_*, and *m_ij_* are the stress tensor, strain tensor, symmetric curvature tensor, and couple stress tensor, respectively; *μ* and *λ* are Lame constants; *l* is the scale parameter of the material that is related to the size effect; *θ_i_* is the rotation vector; *e_ijk_* is the permutation symbol; *E* is the elastic modulus; and *υ* is Poisson’s ratio, respectively.

The Kirchhoff thin-plate theory assumes that the deformation in the middle of the plane is zero. The middle plane is considered the coordinate plane, considering small deflection and ignoring high-order nonlinear terms. Displacement field u_i_ is expressed as [36,37]:

(8) $$u(x,y,z)=-z\frac{\partial w}{\partial x}$$

(9) $$v(x,y,z)=-z\frac{\partial w}{\partial y}$$

(10) w(x,y,z)=w(x,y,t)

Strain components *ε_xx_*, *ε_yy_*, and *γ_xy_* are described using deflection as:

(11) $$\varepsilon_{xx}=\frac{\partial u}{\partial x}=-z\frac{\partial^{2}w}{\partial x^{2}}$$

(12) $$\varepsilon_{yy}=\frac{\partial v}{\partial y}=-z\frac{\partial^{2}w}{\partial y^{2}}$$

(13) $$\gamma_{xy}=\frac{\partial v}{\partial x}+\frac{\partial u}{\partial y}=-z\frac{\partial^{2}w}{\partial x\partial y}$$

The rotation components can be described as:

(14) $$\theta_{x}=\frac{\partial w}{\partial y}$$

(15) $$\theta_{y}=-\frac{\partial w}{\partial x}$$

(16) $$\theta_{z}=0$$

Using Equations (14)–(16) in Equation (3), the nonzero curvature components can be derived as follows:

(17) $$\chi_{xx}=\frac{\partial^{2}w}{\partial x\partial y}$$

(18) $$\chi_{yy}=-\frac{\partial^{2}w}{\partial x\partial y}$$

(19) $$\chi_{xy}=\chi_{yx}=\frac{1}{2}(\frac{\partial^{2}w}{\partial y^{2}}-\frac{\partial^{2}w}{\partial x^{2}})$$

(20) $$\chi_{xz}=\chi_{zx}=\chi_{yz}=\chi_{zy}=\chi_{zz}=0$$

The stress tensor can be described using deflection as follows:

(21) $$\sigma_{xx}=-\frac{Ez}{1-\upsilon^{2}}(\frac{\partial^{2}w}{\partial x^{2}}+\upsilon \frac{\partial^{2}w}{\partial y^{2}})$$

(22) $$\sigma_{yy}=-\frac{Ez}{1-\upsilon^{2}}(\frac{\partial^{2}w}{\partial y^{2}}+\upsilon \frac{\partial^{2}w}{\partial x^{2}})$$

(23) $$\tau_{xy}=\tau_{yx}=-\frac{Ez}{1+\upsilon }\frac{\partial^{2}w}{\partial x\partial y}$$

According to Equation (5), the Lame constant is shown as μ [38,39]; the expression of the couple stress tensor is:

(24) $$m_{xx}=2l^{2}\mu \frac{\partial^{2}w}{\partial x\partial y}$$

(25) $$m_{yy}=-2l^{2}\mu \frac{\partial^{2}w}{\partial x\partial y}$$

(26) $$m_{xy}=m_{yx}=l^{2}\mu \left(\frac{\partial^{2}w}{\partial y^{2}}-\frac{\partial^{2}w}{\partial x^{2}}\right)$$

(27) $$m_{xz}=m_{zx}=m_{yz}=m_{zy}=m_{zz}=0$$

The bending moment and shear force can be written as [40]:

(28) $$M_{x}=\int_{h}\sigma_{xx}zdz+\int_{h}m_{xy}dz=\frac{-Eh^{3}}{12(1-\upsilon^{2})}(\frac{\partial^{2}w}{\partial x^{2}}+\upsilon \frac{\partial^{2}w}{\partial y^{2}})-l^{2}Gh(\frac{\partial^{2}w}{\partial x^{2}}-\frac{\partial^{2}w}{\partial y^{2}})$$

(29) $$M_{y}=\int_{h}\sigma_{xx}zdz-\int_{h}m_{yx}dz=\frac{-Eh^{3}}{12(1-\upsilon^{2})}(\frac{\partial^{2}w}{\partial y^{2}}+\upsilon \frac{\partial^{2}w}{\partial x^{2}})-l^{2}Gh(\frac{\partial^{2}w}{\partial y^{2}}-\frac{\partial^{2}w}{\partial x^{2}})$$

(30) $$M_{xy}=M_{yx}=\int_{h}\sigma_{xy}zdz-\int_{h}m_{xx}dz=\int_{h}\sigma_{yx}zdz+\int_{h}m_{yy}dz==\frac{-Eh^{3}}{12(1-\upsilon^{2})}\frac{\partial^{2}w}{\partial x\partial y}-2l^{2}Gh\frac{\partial^{2}w}{\partial x\partial y}$$

(31) $$Q_{x}=\int_{h}\sigma_{xz}dz$$

(32) $$Q_{y}=\int_{h}\sigma_{yz}dz$$

Using the equilibrium method and ignoring higher-order increments, the relationship between load and bending can be expressed as:

(33) $$\frac{\partial M_{x}}{\partial x}+\frac{\partial M_{yx}}{\partial y}=Q_{x}$$

(34) $$\frac{\partial M_{y}}{\partial y}+\frac{\partial M_{xy}}{\partial x}=Q_{y}$$

(35) $$\frac{\partial Q_{x}}{\partial x}+\frac{\partial Q_{y}}{\partial y}+q=0$$

The bending equilibrium equation of a micro-sized thin plate can be written using Equations (33)–(35) as:

(36) $$\frac{\partial^{2}M_{x}}{\partial x^{2}}+2\frac{\partial^{2}M_{xy}}{\partial x\partial y}+\frac{\partial^{2}M_{y}}{\partial y^{2}}+q=0$$

The components of the bending equilibrium equation in Equation (36) are:

(37) $$\frac{\partial^{2}M_{x}}{\partial x^{2}}=-D(\frac{\partial^{4}w}{\partial x^{4}}+\upsilon \frac{\partial^{4}w}{\partial x^{2}\partial y^{2}})-l^{2}Gh(\frac{\partial^{4}w}{\partial x^{4}}-\frac{\partial^{4}w}{\partial x^{2}\partial y^{2}})$$

(38) $$\frac{\partial^{2}M_{y}}{\partial y^{2}}=-D(\frac{\partial^{4}w}{\partial y^{4}}+\upsilon \frac{\partial^{4}w}{\partial x^{2}\partial y^{2}})-l^{2}Gh(\frac{\partial^{4}w}{\partial y^{4}}-\frac{\partial^{4}w}{\partial x^{2}\partial y^{2}})$$

(39) $$\frac{\partial^{2}M_{xy}}{\partial x\partial y}=-D(1-\upsilon )\frac{\partial^{4}w}{\partial x^{2}\partial y^{2}}-2l^{2}Gh\frac{\partial^{4}w}{\partial x^{2}\partial y^{2}}$$

where D is the flexural rigidity. The expression is given by:

(40) $$D=\frac{Eh^{3}}{12(1-\upsilon^{2})}$$

Using Equations (37)–(39) into Equation (36), the bending equilibrium equation based on the modified couple theory is expressed as:

(41) $$(D+l^{2}Gh)\nabla^{4}w=q$$

The classical bending equilibrium equation [41,42,43,44] based on the Kirchhoff thin-plate theory is:

(42) $$D\nabla^{4}w=q$$

Comparing the bending equilibrium equation of the classical theory (Equation (41)) and the modified couple stress theory (Equation (42)) and simply replacing the bending stiffness in the CT, the MCST can be converted:

(43) $$\left\{ \begin{array}{l} D=\frac{Eh^{3}}{12(1-\upsilon^{2})}\\ D_{1}=\frac{Eh^{3}}{12(1-\upsilon^{2})}+l^{2}Gh \end{array}\right.$$

When scale parameter *l* is 0, the MCST can be reduced to the result of the CT. Above, *D*_1_ is the bending stiffness considering the scale effect based on the MCST, and *D*_2_ is the equivalent bending stiffness (EBS) based on the MCST, which can be defined as:

(44) $$D_{2}=\frac{12(1-\upsilon^{2})}{Eh}D_{1}$$

*D*_2_ is achieved as follows:

(45) $$D_{2}=h^{2}+6(1-\upsilon )l^{2}$$

### 2.1. Bending Problem

Given a thin plate as in Figure 1 that is only subjected to lateral load *q*(*x*,*y*), the equilibrium equation for bending is:

(46) $$D(1+\frac{6l^{2}(1-\upsilon )}{h^{2}})\nabla^{4}w=q(x,y)$$

All thin-plate edges are simply supported. The deflection and bending moment of the boundary conditions along all edges of the thin plate are zero. The boundary conditions for simply supported edges can be written as:

(47) $$w(0,y)=0\;M_{xx}(0,y)={\left(\frac{\partial^{2}w}{\partial x^{2}}\right)}_{x=0}=0\;w(a,y)=0\;M_{xx}(a,y)={\left(\frac{\partial^{2}w}{\partial x^{2}}\right)}_{x=a}=0\;w(x,0)=0\;M_{xx}(x,0)={\left(\frac{\partial^{2}w}{\partial x^{2}}\right)}_{y=0}=0\;w(x,b)=0\;M_{xx}(x,b)={\left(\frac{\partial^{2}w}{\partial x^{2}}\right)}_{y=b}=0$$

Based on the Navier method, the deflection function can be defined as a double trigonometric series. The Navier method is the most effective method to solve the bending problem of simply supported thin plates. This method is invalid when the boundary conditions change. The differential quadrature (DQM) and generalized differential quadrature methods (GDQM) converge quickly and accurately, which can be applied to any boundary condition [45,46,47,48]:

(48) $$w\left(x,y\right)=\sum_{m=1}^{\infty }\sum_{n=1}^{\infty }A_{mn}\sin\frac{m\pi x}{a}\sin\frac{n\pi y}{b}$$

Equation (48) can satisfy all boundary conditions, where *A_mn_* is the Fourier coefficient, and *a* and *b* are the length and width of thin plates, respectively. Similarly, external lateral load *q*(*x*,*y*) can be defined in the same form:

(49) $$q\left(x,y\right)=\sum_{m=1}^{\infty }\sum_{n=1}^{\infty }C_{mn}\sin\frac{m\pi x}{a}\sin\frac{n\pi y}{b}$$

According to the Fourier series expansion formula, coefficient *C_mn_* can be obtained as:

(50) $$C_{mn}=\frac{4}{ab}\int_{0}^{a}\int_{0}^{b}q\sin\frac{m\pi x}{a}\sin\frac{n\pi y}{b}dxdy$$

The simplified equilibrium equation for bending can be obtained using Equations (48) and (49) into Equation (46):

(51) $$\pi^{4}\left(D+l^{2}Gh\right)\sum_{m=1}^{\infty }\sum_{n=1}^{\infty }{\left(\frac{m^{2}}{a^{2}}+\frac{n^{2}}{b^{2}}\right)}^{2}A_{mn}\sin\frac{m\pi x}{a}\sin\frac{n\pi y}{b}=\sum_{m=1}^{\infty }\sum_{n=1}^{\infty }C_{mn}\sin\frac{m\pi x}{a}\sin\frac{n\pi y}{b}$$

The expansion terms of the series on both sides of the equation are equal to ensure that the two series are equal. Fourier coefficient *A_mn_* is achieved as follows:

(52) $$A_{mn}=\frac{4\int_{0}^{a}\int_{0}^{b}q\sin\frac{m\pi x}{a}\sin\frac{n\pi y}{b}dxdy}{\left(D+l^{2}Gh\right)\pi^{4}ab{\left(\frac{m^{2}}{a^{2}}+\frac{n^{2}}{b^{2}}\right)}^{2}}$$

When the lateral load is uniformly distributed, *q*(*x*,*y*) = *q*_0_. Coefficients *C_mn_* and *A_mn_* can be described for odd values of both *m* and *n* as:

(53) $$C_{mn}=\frac{16q_{0}}{mn\pi^{2}}$$

(54) $$A_{mn}=\frac{C_{mn}}{\left(D+l^{2}Gh\right)\pi^{4}{\left(\frac{m^{2}}{a^{2}}+\frac{n^{2}}{b^{2}}\right)}^{2}}$$

Using Equation (54) in Equation (48), the deflection can be achieved as:

(55) $$w=\frac{16q_{0}}{\pi^{6}\left(D+l^{2}Gh\right)}\sum_{m=1,3,5}^{\infty }\sum_{n=1,3,5}^{\infty }\frac{\sin\frac{m\pi x}{a}\sin\frac{n\pi y}{b}}{mn{\left(\frac{m^{2}}{a^{2}}+\frac{n^{2}}{b^{2}}\right)}^{2}}$$

Maximum deflection that occurs at the midpoint of the thin plate can be expressed as:

(56) $$w_{\max}=\frac{16q_{0}}{\pi^{6}\left(D+l^{2}Gh\right)}\sum_{m=1,3,5}^{\infty }\sum_{n=1,3,5}^{\infty }\frac{(-1{)}^{\frac{m+n}{2}-1}}{mn{\left(\frac{m^{2}}{a^{2}}+\frac{n^{2}}{b^{2}}\right)}^{2}}$$

Under the conditions that *a* = *b*, the maximum deflection of the square thin plate can be written as:

(57) $$w_{\max}=\frac{16q_{0}a^{4}}{\pi^{6}\left(D+l^{2}Gh\right)}=0.00416\frac{q_{0}a^{4}}{\left(D+l^{2}Gh\right)}$$

where $w^{\sim }{}_{\max}$ is the maximum equivalent deflection of the square plate, which can be defined as follows:

(58) $$w^{\sim }{}_{\max}=\frac{w_{\max}}{q_{0}a^{4}}*\frac{Eh}{12(1-\upsilon^{2})}=\frac{0.00416}{h^{2}+6(1-\upsilon^{2})l^{2}}$$

### 2.2. Buckling Problem

**Figure 2:**
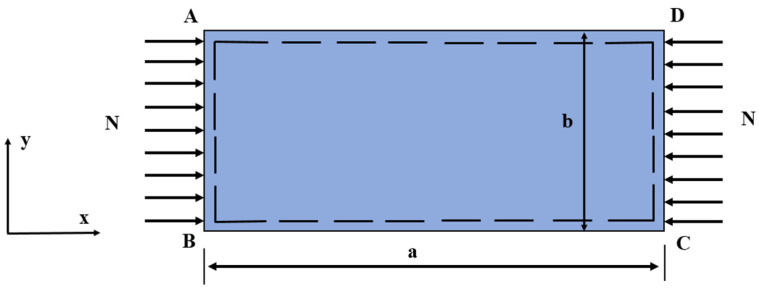
Loading and geometry of the thin-plate buckling problem.

All thin-plate edges are simply supported and only subjected to uniaxial compression load Nx. The schematic diagram is shown in Figure 2.

The equilibrium equation for buckling is:

(59) $$(D+l^{2}Gh)\nabla^{4}w+N_{x}\frac{\partial^{2}w}{\partial x^{2}}=0$$

The deflection and moment of the boundary conditions along all edges of the thin plate are zero. The boundary conditions for simply supported edges can be expressed as:

(60) $$w=0\;\mathrm{on}\;\mathrm{ABCD}\;M_{n}=0\;\mathrm{on}\;\mathrm{ABCD}$$

The solution is sought as a product of two harmonic functions:

(61) $$w\left(x,y\right)=w_{0}\sin\frac{m\pi x}{a}\sin\frac{n\pi y}{b}$$

where *m* is the number of half-sine waves in the *x*-direction, *n* is the number of half-sine waves in the *y*-direction, and *w*_0_ is a deflection-related constant. Equation (61) can satisfy all corresponding boundary conditions.

Using Equation (61) in Equation (59) can achieve:

(62) $$\left\{(D+l^{2}Gh)\left[{\left(\frac{m\pi }{a}\right)}^{4}+2{\left(\frac{m\pi }{a}\right)}^{2}{\left(\frac{n\pi }{b}\right)}^{2}+{\left(\frac{n\pi }{b}\right)}^{4}\right]-N_{x}{\left(\frac{m\pi }{a}\right)}^{2}\right\}w_{0}\sin\frac{m\pi x}{a}\sin\frac{n\pi y}{b}=0$$

The differential equation is satisfied for all values of (*x*,*y*) if the coefficients satisfy:

(63) $$N_{x}=\left(D+l^{2}Gh\right){\left(\frac{\pi a}{m}\right)}^{2}{\left[{\left(\frac{m}{a}\right)}^{2}+{\left(\frac{n\pi }{b}\right)}^{2}\right]}^{2}$$

Considering a simple condition, when *n* = 1, Equation (63) can be written as:

(64) $$N_{x}{}_{c}=k_{c}\frac{\pi^{2}}{b^{2}}\left(D+l^{2}Gh\right)$$

where *N_xc_* is the critical buckling load and kc is the buckling coefficient, which can be defined as [33]:

(65) $$k_{c}={\left(\frac{mb}{a}+\frac{a}{mb}\right)}^{2}$$

It can be seen from Equations (64) and (65) that scale parameter *l* only affects the critical buckling load and has no effects on the buckling coefficient. This means that the scale effect does not affect the buckling topography.

$N^{\sim }{}_{xc}$ is the equivalent critical buckling load of the thin plate and is defined as follows:

(66) $$N^{\sim }{{}_{x}}_{c}=N_{x}{}_{c}\frac{b^{2}}{\pi^{2}}\frac{12(1-\nu^{2})}{Eh}={\left(\frac{mb}{a}+\frac{a}{mb}\right)}^{2}\left[h^{2}+6\left(1-\upsilon \right)l^{2}\right]$$

### 2.3. Free Vibration Problem

The nonhomogeneous motion equation of isotropic thin plates considering the scale effect can be expressed as:

(67) $$\left(D+l^{2}Gh)\nabla^{4}w+\rho h\frac{\partial^{2}w}{\partial t^{2}}=p(x,y,t\right)$$

where *ρ* is the density of the thin plate and *p*(*x*,*y*,*t*) is the dynamic load. Considering the problem of free vibration, *p*(*x*,*y*,*t*) is equal to zero. Equation (67) can be achieved as follows:

(68) $$(D+l^{2}Gh)\nabla^{4}w+\rho h\frac{\partial^{2}w}{\partial t^{2}}=0$$

To solve Equation (68), we define *w* as follows:

(69) w=T(t)∗W(x,y)

where *T*(*t*) can be assumed as follows:

(70) T(t)=Asinωt+Bcosωt

where *ω* is the thin-plate frequency, *A* and *B* are constant, and *t* is time. All thin-plate edges are simply supported. The mode shape expression (*W*(*x*,*y*)) in Equation (69) can be written as:

(71) $$W\left(x,y\right)=w_{0}\sin\frac{m\pi x}{a}\sin\frac{n\pi y}{b}$$

where *m* is the number of half-sine waves in the *x*-direction, *n* is the number of half-sine waves in the *y*-direction, *w*_0_ is a deflection-related constant, and *a* and *b* are the length and width of the thin plates, respectively. Using Equation (69) in Equation (68), we can obtain:

(72) $$(D+l^{2}Gh)\pi^{4}(\frac{m^{2}}{a^{2}}+\frac{n^{2}}{b^{2}}{)}^{2}-\omega^{2}\rho h=0$$

When *m* and *n* are given, the value of natural frequency *ω_mn_* can be obtained as follows:

(73) $$\omega_{mn}{}^{2}=\frac{(D+l^{2}Gh)\pi^{4}}{\rho h}(\frac{m^{2}}{a^{2}}+\frac{n^{2}}{b^{2}}{)}^{2}$$

## 3. Numerical Simulation Results and Analysis

In this section, the bending, buckling, and free vibration of Cu thin plates under simply supported boundary conditions are investigated based on the modified couple stress theory. The Cu material and geometric parameters are as follows:

E = 110 GPa, υ = 0.35, a = b = 200 μm, ρ = 8900 kg/m^3^, q_0_ = 0.1 μN/μm

**Figure 3:**
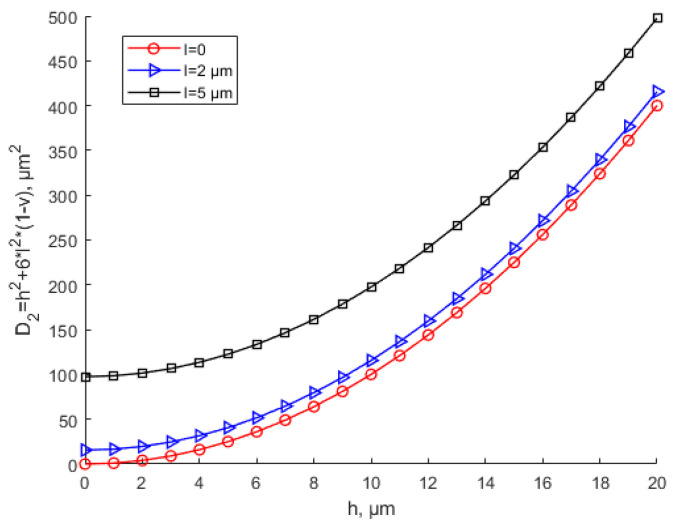
Effects of the scale parameter on equivalent bending stiffness based on CT and MCST.

**Figure 4:**
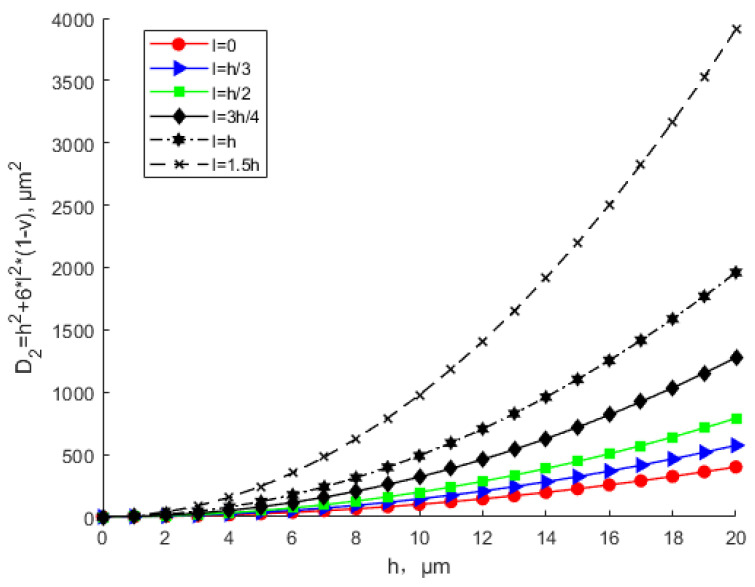
Effects of the scale parameter about *h* on equivalent bending stiffness based on CT and MCST.

It can be seen from Figure 3 and Figure 4 that with the same thickness, as scale parameter l increases, the equivalent stiffness tends to increase. The equivalent stiffness base on the MCST is larger than that based on the CT for the same thickness. For the same scale parameter, as the thickness increases, the equivalent stiffness increases. Figure 4 shows that when l = 1.5 h, with the increase in the thickness, the equivalent stiffness deviates sharply from the that based on the CT. In this situation, the scale effect cannot be ignored and plays a leading role.

**Figure 5:**
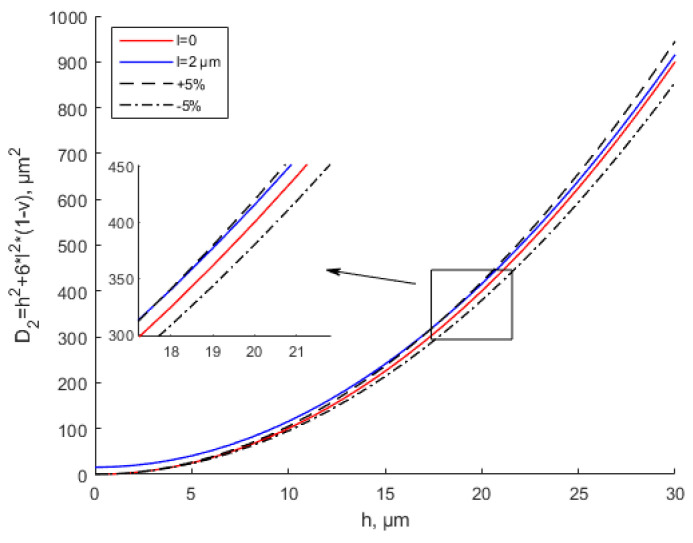
Modified couple stress and classical equivalent bending stiffness of thin plates shown with a small error when *l* = 2 μm.

**Figure 6:**
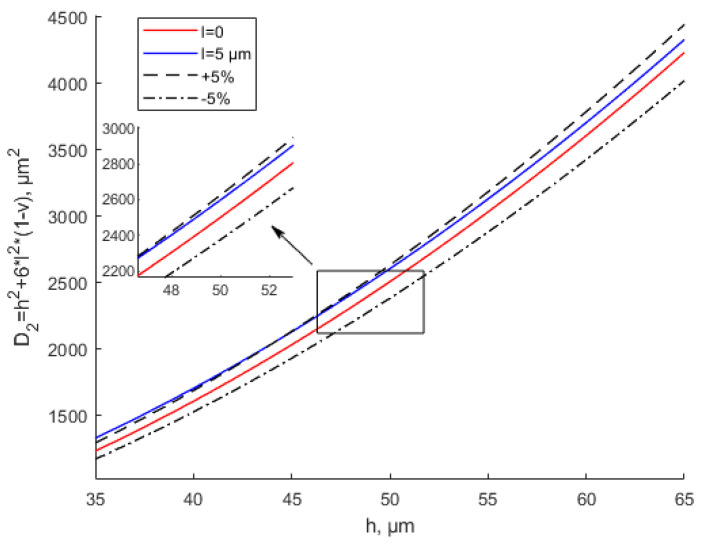
Modified couple stress and classical equivalent bending stiffness of thin plates shown with a small error when *l* = 5 μm.

Many experiments have verified the effectiveness of the modified couple stress theory [35,49,50]. However, the literature has not given a specific scope of application based on the MCST. In Figure 5 and Figure 6, ±5% represent the deviation values of the equivalent stiffness based on the CT. When *l* = 2 μm, the equivalent bending stiffness increases with the increase in the thickness. When *h* ≥ 10*l*, the MCST (modified couple stress theory) is close to the CT (classical theory). The deviation between the equivalent stiffness values based on the MCST and CT is within ±5%. It can be considered that the scale effect is negligible at this time. It shows great agreement with the study by Li et al. [35]. The scale effect parameter of Ti is 0.775 μm based on the MCST. When thickness *h* ≥ 10*l*, As the thickness increases, there are no changes in the dimensionless frequency and dimensionless bending rigidity. In Figure 5, when the thickness is between 0 and 20 μm, the deviation between the equivalent stiffness values based on the MCST and CT is greater than +5%. The scale effect cannot be ignored at this time. Similarly, in Figure 6, when the thickness is less than 50 μm, the deviation between the equivalent stiffness values based on the MCST and CT is greater than +5%. The scale effect is of great significance at this time.

**Figure 7:**
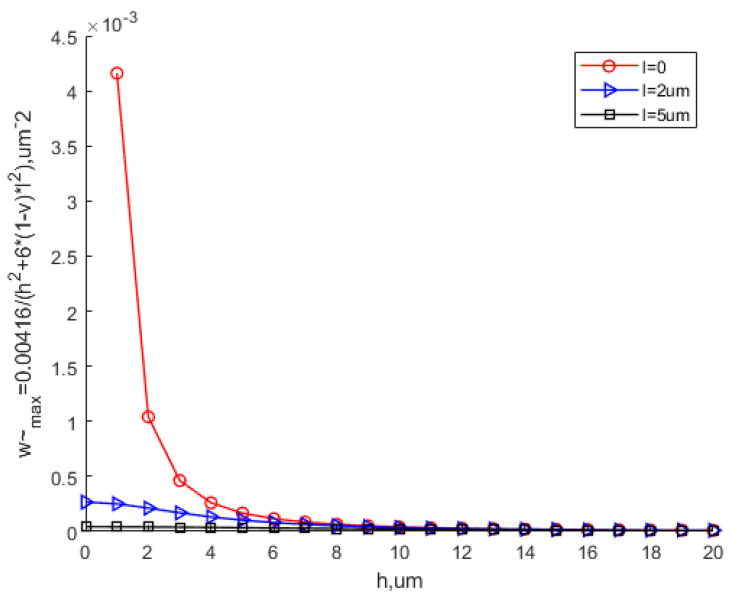
Effect of the scale parameter on the equivalent maximum deflection.

Figure 7 shows that the scale parameter can greatly reduce the equivalent maximum deflection, especially when the thickness is small. The equivalent maximum deflection decreases with the increase in the thickness. With the continuous increase in the thickness, the equivalent maximum deflection based on the MCST is close to that based on the CT. The scale effect can be ignored at this time.

**Figure 8:**
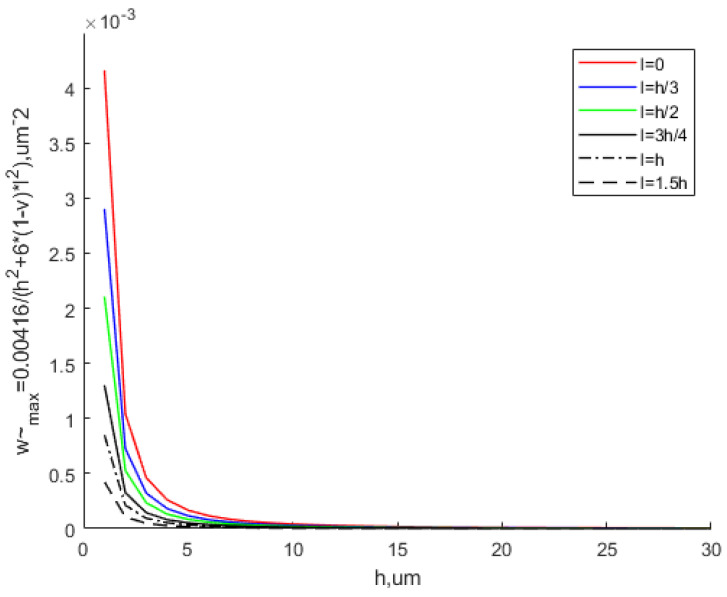
Variation in the equivalent maximum deflection with various values of the scale parameter.

Figure 8 shows the variation in the equivalent maximum deflection with different scale parameters. It can be seen that with the same thickness, as the scale parameter increases, the equivalent maximum deflection decreases, especially when the thickness is small. As the thickness continues to increase, the equivalent maximum deflection tends to be the same. This means that the scale effect does not work in this situation.

**Figure 9:**
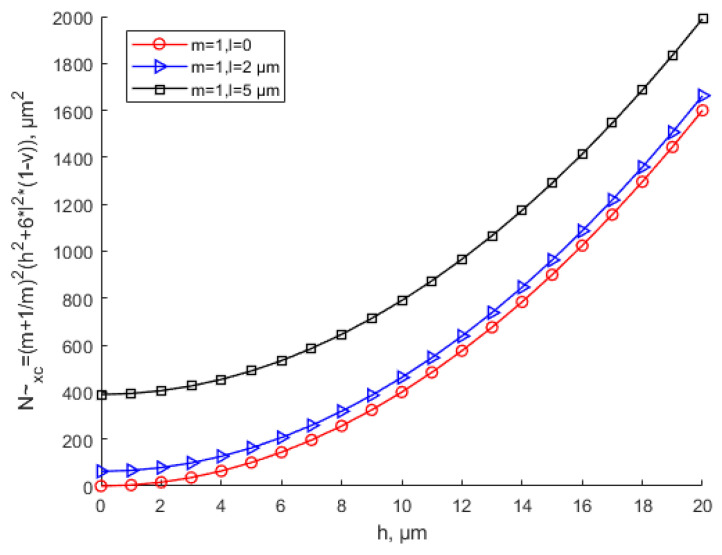
Variation in the equivalent critical buckling load with various values of the scale parameter.

**Figure 10:**
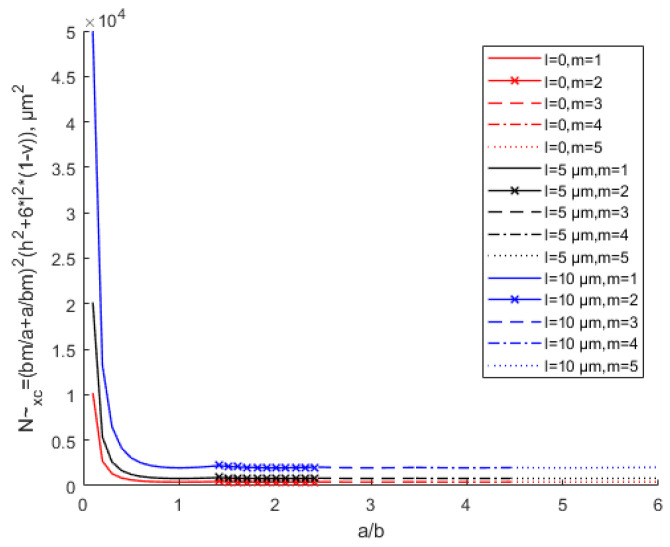
Variation in the equivalent critical buckling load with various values of the scale parameter and different aspect ratios when *h* = 10 μm.

Figure 9 shows that the equivalent critical buckling load increases with the increase of the scale effect parameter. Figure 10 shows the influence of the aspect ratio and scale parameters on the equivalent critical buckling load when *h* = 10 μm. In the case of *n* = 1, different aspect ratios correspond to different *x*-direction half-sine wave numbers *m*. The scale effect does not affect the buckling coefficient. This means that the scale effect does not change the buckling topography, which only increases the equivalent critical buckling load with the scale parameter increase in the case of the same aspect ratio. It can also be seen that with the same thickness and aspect ratio, the equivalent critical buckling load increases with the increase in the scale parameter. With the same scale parameter, the equivalent buckling load decreases with the increase in the aspect ratio, especially when the aspect ratio is small. As the aspect ratio keeps increasing, the equivalent critical buckling load tends to be the minimum constant value. The value of the minimum equivalent critical buckling load is $N^{\sim }{{}_{x}}_{c\min}=4\left[h^{2}+6\left(1-\upsilon \right)l^{2}\right]$.

**Figure 11:**
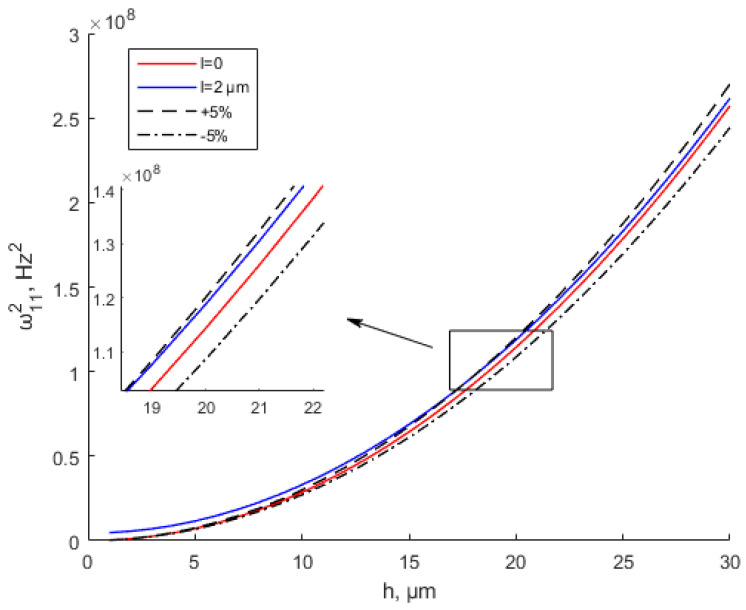
Modified couple stress and classical natural frequency of thin plates shown with small deviation when *l* = 2 μm.

**Figure 12:**
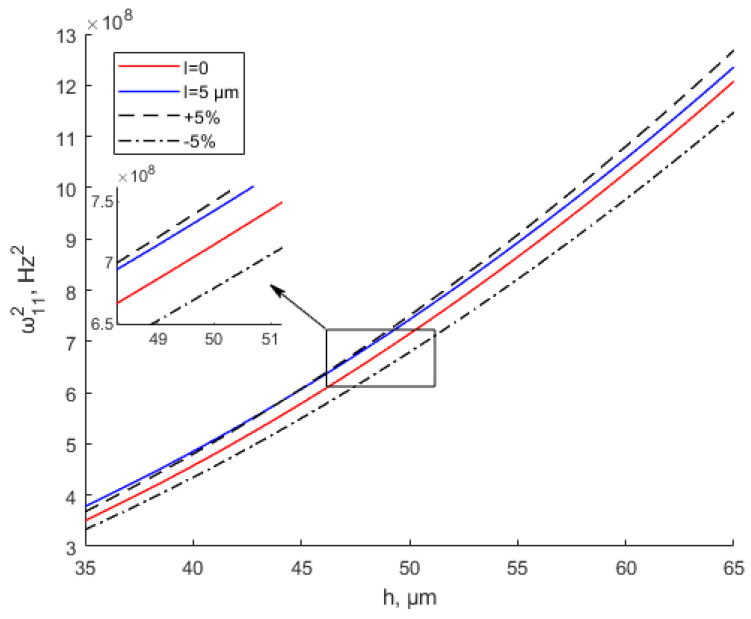
Modified couple stress and classical natural frequency of thin plates shown with small deviation when *l* = 5 μm.

**Figure 13:**
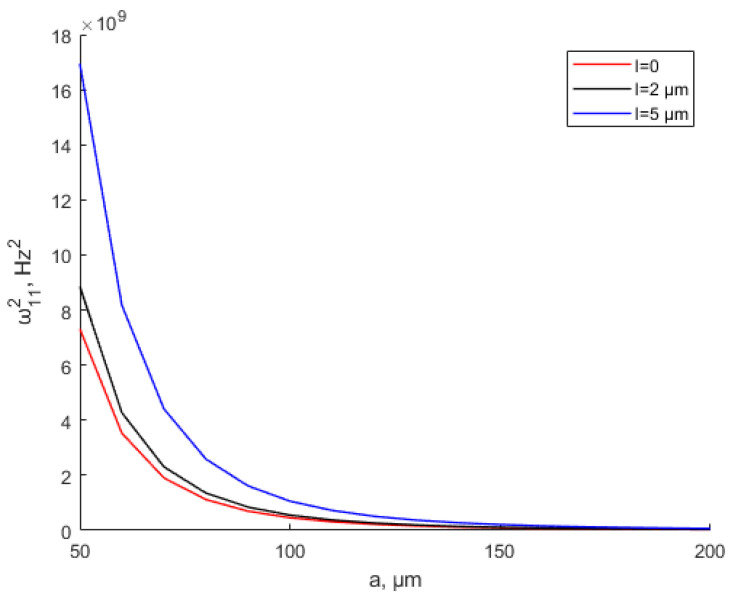
Variation in the natural frequency with various values of the scale parameter when *h* = 10 μm.

Figure 11 and Figure 12 show the variation in the natural frequency with various values of the scale parameter and thickness when *a* = *b* = 200 μm. As shown in the graph, the natural frequency increases with the thickness increase. When the thickness is the same, the natural frequency increases with the increase in the scale parameter. It shows the same conclusion as the study by Mohsen Namvar et al. [49]. The scale effect increases the flexural rigidity and decreases static plate deflections, leading to increased values of the natural frequency. Figure 12 and Figure 13 also show that when *h* > 10*l*, the scale effect can be ignored. The natural frequency values based on the MSCT and CT show a small error within ±5% deviation when *h* > 10*l*. The study by Bo Zhang et al. [51] can prove this conclusion. The study [51] shows that when *h*/*l* > 10, the fundamental natural frequency is almost equal between the CT and MCST.

Figure 13 shows the natural frequency changes with different scale parameters and different square thin-plate lengths when *h* = 10 μm. The results show that when the square thin-plate length is small, the natural frequency increases with the increase in the scale parameter. As the square thin-plate length keeps increasing, the natural frequency drops and tends to be the smallest constant value.

Since natural frequency results for micro plates of Cu materials are not available in the open literature, considering the micro plate is made of epoxy, the material properties can be seen in Table 1. The natural frequencies of homogeneous epoxy square plates under simply supported conditions are shown in Table 2. Present I shows the natural frequencies without considering scale effects, and Present II demonstrates the influence of scale effects. The natural frequencies considering the scale effect are larger than those obtained without considering it. Since Present II only considers small deflection linear vibrations, ignoring higher-order nonlinear terms, this results in natural frequency values lower than those found by Choi et al. [52] and Ke et al. [53].

## 4. Conclusions

The impact of the scale effect on the mechanical properties of thin plates is studied in this paper. Based on the modified couple stress theory combined with the Kirchhoff thin-plate theory, the force equilibrium method is used to derive the governing equations. The bending stiffness of the plate is redefined with the scale effect, called the EBS model, and the influence of the scale effect on the EBS is studied. Analytical solutions for bending, buckling, and free vibration are obtained. The influences of scale effect parameters on the bending, free vibration, and buckling topography of micro-scale thin plates are discussed in detail. The conclusions are as follows:

(1) The scale effect only makes a significant difference when the thickness is very small. Specifically, the difference is obvious when the thickness is less than 10 times the scale effect parameter. When the thickness is greater than 10 times the scale effect parameter, the MCST and CT theoretical results are close. The scale effect can be ignored in this situation. The scale effect parameter of the material is experimentally measurable and unique. Thus, the applicable scope of the scale effect under the modified couple stress theory is determined;
(2) The scale effect changes the deflection and critical buckling loads by changing the EBS. However, it does not affect the buckling topography. The stiffness hardening of Cu thin plates is due to the scale effect. With the increase in scale effect parameter *l*, the value of the EBS increases, resulting in the decreases in the equivalent deflection and the increases in the equivalent critical buckling load;
(3) For the scale effect of the free vibration response, with the increase in scale effect parameter *l*, the micro-scale thin-plate natural frequency increases. The natural frequency decreases as the length of the square micro-scale thin plate increases and finally stabilizes. The natural frequencies in this study are smaller than those in other nonlinear vibration analyses due to the neglect of higher-order terms.

It is of great significance to consider scale effect parameters for micro-scale plates. According to the EBS model, both the thickness and scale effect parameters increase the value of the EBS; the scale effect plays a dominant role when the thickness is small, and the effect of the thickness on the EBS is greater when the thickness is larger. A large value of the EBS represents a stiffer plate, which results in reduced deflection, increased critical buckling load, and increased free vibration frequency. However, the buckling topography is only determined by the plate size, so the scale effect does not affect it. This can be used for the design, fabrication, and application of MEMS/NEMS micro-scale-structured devices.

## Figures and Tables

**Table 1:** Material properties of epoxy.

Material	*E*	*υ*	*ρ*	*l*
Epoxy [53]	1.44 GPa	0.38	1220 kg/m^3^	17.6 μm

**Table 2:** Comparison of the natural frequencies (MHz) of homogeneous square plates (*h* = 2*l*).

**Mode**	*a*/*h*	Choi et al. [52]	Ke et al. [53]	Present I (*l* = 0)	Present II (*l* = 17.6 μm)
1	5	1.6289	1.4701	0.7605	1.056
	10	0.4170	0.4042	0.1901	0.2641
	20	0.1049	0.1040	0.0475	0.0661
	30	0.0467	0.0465	0.0221	0.0293

## Data Availability

Not applicable.
